# Feed additives for the control of post-weaning *Streptococcus suis* disease and the effect on the faecal and nasal microbiota

**DOI:** 10.1038/s41598-020-77313-6

**Published:** 2020-11-23

**Authors:** Florencia Correa-Fiz, Carlos Neila-Ibáñez, Sergio López-Soria, Sebastian Napp, Blanca Martinez, Laia Sobrevia, Simon Tibble, Virginia Aragon, Lourdes Migura-Garcia

**Affiliations:** 1 IRTA, Centre de Recerca en Sanitat Animal (CReSA, IRTA-UAB), Campus de la Universitat Autònoma de Barcelona, 08193 Bellaterra, Spain; 2 OIE Collaborating Centre for the Research and Control of Emerging and Re-Emerging Swine Diseases in Europe (IRTA-CReSA), Bellaterra, Barcelona Spain; 3 ASN SL, Calle de Murcia, PL Fraga, 22520 Huesca, Spain

**Keywords:** Antimicrobials, Microbial communities

## Abstract

Medicated feed is a common strategy to control the occurrence of *Streptococcus suis* disease in swine production, but feed additives may constitute an alternative to metaphylaxis. In a farm with post-weaning *S. suis* disease, the following additives were tested: lysozyme (Lys), medium chain fatty acids plus lysozyme (FA + Lys), FA plus a natural anti-inflammatory (FA + antiinf) and amoxicillin (Amox). During the course of the study, FA + antiinf and Amox groups showed lower prevalence of clinical signs compatible with *S. suis* disease than the rest of the groups. Piglets from the FA + antiinf group showed high diversity and richness in their nasal and faecal microbiota. Diet supplements did not have major effects on the faecal microbiota, where the genus *Mitsuokella* was the only differentially present in the FA + Lys group. In the nasal microbiota, piglets from FA + antiinf presented higher differential abundance of a sequence variant from *Ruminococcaceae* and lower abundance of an unclassified genus from *Weeksellaceae*. In general, we detected more significant changes in the nasal than in the feacal microbiota, and found that parity of the dams affected the microbiota composition of their offspring, with piglets born to gilts exhibiting lower richness and diversity. Our results suggest that additives could be useful to control post-weaning disease when removing antimicrobials in farms.

## Introduction

During the last decades, antimicrobials have been used in human and veterinary medicine for the treatment of bacterial infectious diseases. The excessive use of these drugs has increased the selective pressure causing the emergence of multi-drug resistant bacteria in both, human and veterinary medicine^1^. For many years, conventional farming and in particular, pig production has routinely controlled the occurrence and transmission of diseases during critical periods of the rearing cycle by medicating the whole herd, including healthy animals^2^. Weaning represents one of these critical periods with the highest risk of enteric and respiratory diseases, commonly requiring the use of antimicrobials^3^. After farrowing, litters are normally mixed and young piglets of approximately 21 days old, with an immature immune system, have to adapt to the new environment in combination with a change of diet from milk to dried feed. This situation leaves piglets vulnerable to infections caused by multiple pathogens, including *Streptococcus suis*.

*S. suis* is an early colonizer of the upper respiratory track (tonsils and nasal cavities) of young piglets. Virulent strains can cause severe outbreaks after weaning, when maternal antibodies are at the lowest level^4^. Meningitis and septicaemia are the most severe manifestation of the disease, causing up to 30% mortality rates^5^, but endocarditis, pneumonia and polyarthritis have also been reported^6^. Diagnostic at the farm is generally performed by the veterinarian based on clinical signs and macroscopic lesions.

For many years, the first option to control the emergence and spread of *S. suis* has been the prescription of feed supplemented with antimicrobials, generally a beta-lactam such as penicillin or amoxicillin^7^. Nowadays, due to the emergence of multi-drug resistant bacteria, this use has to be well justified, and national action plans are focused on implementing antimicrobial stewardship strategies to minimise the consumption of antimicrobials in food producing animals^8^ Different strategies have been suggested to comply with this new framework and reduce the use of antimicrobials. Optimization of nutritional programs during the post-weaning period can favour the growth of beneficial bacteria resulting in a positive effect in the immune system of the animals and their health^9,10^.

With the objective of removing the use of amoxicillin, this study aimed to assess the effect of three different combinations of feed additives during post-weaning on the occurrence of disease caused by *S. suis*. Clinical signs, faecal and nasal microbiota composition together with production parameters were compared among groups of piglets fed with the different additives and those treated with amoxicillin.

## Results

### Clinical status and productive performance

A total of 569 twenty-one days-old piglets were divided into 5 treatment groups (lysozyme (Lys), medium chain fatty acids and lysozyme (FA + Lys), FA and a natural anti-inflammatory (FA + antiinf), amoxicillin (Amox) and negative control). One week before weaning, the average weight per animal in each group was 4 kg. During the course of the study, 135 animals were removed from the five treatment groups due to different clinical signs (Table 1). The majority of the piglets removed (60.7%) exhibited clinical signs compatible with *S. suis* disease*,* such as limping or nervous signs. Coughing and wasting were also detected but with less frequency, 20.3% and 13.7% in FA + antiinf and control groups, respectively.

**Table 1:** Number of animals that needed to be removed from each of the treatment groups including clinical signs compatible with infection caused by *S. suis*, and prevalence of disease in each treatment group.

Treatments	Initial number of animals per group	Final number of animals per group	*S. suis*-like (limping and nervous) signs	Prevalence of *S. suis*-like clinical signs	Number of animals removed^a^	Prevalence of disease
Lysozyme	113	89	20	17.7	24	21.2
FA + lys	110	90	14	12.7	20	18.2
Negative control	112	73	25	22.3	39	34.8
Amoxicillin	115	93	11	9.6	22	19.1
FA + antiinf	119	89	11	9.2	30	25.2
Total	569	434	82	14.4	135	23.7

^a^Includes clinical sings compatible with *S. suis* (limping and nervous signs), coughing, wasting and diarrhea.

The differences in the prevalence of clinical signs compatible with *S. suis* (*S. suis*-like) in the FA + Lys and FA + antiinf groups (12.7% and 9.2%, respectively) compared with the prevalence in the amoxicillin group (9.6%) (Table 1), were not statistically significant (*P* = 0.45 and *P* = 0.93, respectively). In the case of the Lys group, the prevalence of *S. suis*-like clinical signs was higher (17.7%), and compared with the prevalence in the Amox group, the difference was close to being statistically significant (*P* = 0.07). On the other hand, comparisons of the prevalences of *S. suis*-like clinical signs in the treatment groups with the prevalence in the negative control group (22.3%) (Table 1), indicate that the differences were statistically significant for the FA + Lys group (prevalence = 12.7%; *P* = 0.06) and for the FA + antiinf group (prevalence = 9.2%; *P* = 0.006), but not for the Lysozyme group (prevalence = 17.7%; *P* = 0.39).

Throughout the study, 24% of the animals (4, 6, 14, 11 and 19 animals from the Lys, FA + lys, control, Amox and FA + antiinf groups, respectively) were removed due to the presence of any clinical sign. The prevalence of diseases in the Lys (21.2%), FA + Lys (18.2%) and FA + antiinf (25.2%) groups were not significantly different (*P* = 0.69, *P* = 0.86 and *P* = 0.26, respectively) compared to the prevalence in the Amox group (19.1%) (Table 1). Finally, comparisons of the prevalence of diseases in the groups fed with additives with the prevalence in the negative control group (34.8%, Table 1), indicated that the differences were statistically significant for the Lys group (prevalence = 21.2%; *P* = 0.02) and for the FA + Lys group (prevalence = 18.2%; *P* = 0.005), but not for the FA + antiinf group (prevalence = 25.2%; *P* = 0.11).

**Figure 1:**
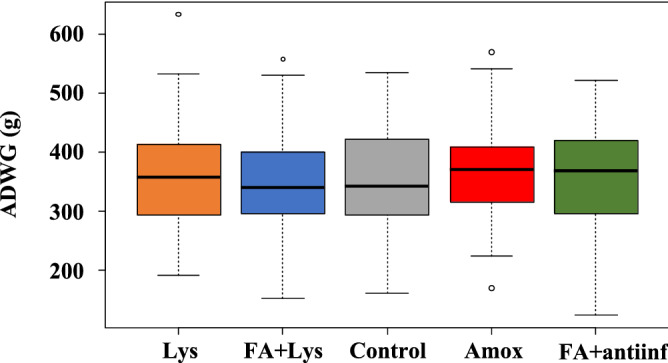
Average daily weight gain (ADWG) of piglets fed with different additives. Each boxplot corresponds to one treatment group: *Lys* lysozyme, *FA + lys* medium chain fatty acids and lysozyme, *C* control, with no additives, *Amox* amoxicillin, *FA + antiinf* medium chain fatty acids and a natural antiinflammatory. Dotted lines represent the standard deviation and outliers are indicated with white circles.

The average daily weight gain by the end of the transition-period in the different treatment groups was between 305.2 and 320.1 g. The Shapiro–Wilk test indicated that weights in the different groups were normally distributed and the ANOVA test demonstrated no significant differences in average daily weight gain (ADWG) among the five treatment groups (*P* = 0.67, Fig. 1).

### Occurrence of *S. suis* and *Glaesserella parasuis* in the farm

Three animals from the non-treated control, Amox and AF + antiinf groups were sacrificed. The post-mortem examination showed that the piglets from the control and FA + antiinf groups exhibited lesions compatible with *S. suis* or *G. parasuis* infection. *S. suis* was confirmed as the cause of the disease at the farm, since a serotype 2 strain was isolated from the lesions of these two animals, confirming the circulation of the pathogen during the study period. *G. parasuis* was not isolated from any of the lesions from the euthanised piglets. The two *S. suis* isolates obtained from the lesions were susceptible to penicillin, ampicillin and ceftiofur. Both of them exhibited the same resistance profile, being phenotypically resistant to chlortetracycline (MIC ≥ 16), oxytetracycline (MIC ≥ 16), clindamycin (> 16), neomycin (MIC ≥ 32), tylosin tartrate (MIC > 32) and tulathromycin (MIC > 64). These two isolates showed the same fingerprinting profile by Enterobacterial Repetitive Intergenic Consensus (ERIC) PCR.

PCR performed on nasal swabs taken at weaning (D0) from 123 piglets out of the subset of 125 (2 nasal swabs were mislabeled), demonstrated the presence of *S. suis* in the nasal cavities of 121 piglets. The two *S. suis*-negative piglets were from different litters. On D43, 102 individual piglets of the subset of 125 still included in the study were swabbed before departure to the finishing farm, and only one was negative for the presence of *S. suis*. This animal belonged to the group treated with amoxicillin, and was different to the two negative animals detected during nursing. No association could be found between the presence of *S. suis* in the nasal samples and the treatment groups. For *G. parasuis*, the initial prevalence of virulent strains based on the 123 piglets analysed by PCR was 37.2%. This prevalence increased at the end of the nursery period, with 98 animals carrying virulent strains out of the 102 analysed (96.1%). This is the expected dynamics of nasal colonization in piglets by *G. parasuis*, with increasing occurrence of *G. parasuis* with age during the nursery period. No association was detected between treatment group and the presence of *G. parasuis* virulent strains.

### Changes in faecal and nasal microbiota composition through time

The V3-V4 region of the 16S rRNA gene was analysed to unravel the microbial composition in both faeces and nasal cavities of the pigs under treatment. Faecal content and nasal swabs taken from 12 piglets at weaning (D0, before treatment) and 10 piglets from each group at 21 days-post-treatment (D21) were included in the analysis. After quality control and filtering steps, the mean frequency of sequences for rectal and nasal samples were 44,184 and 84,429, respectively.

**Figure 2:**
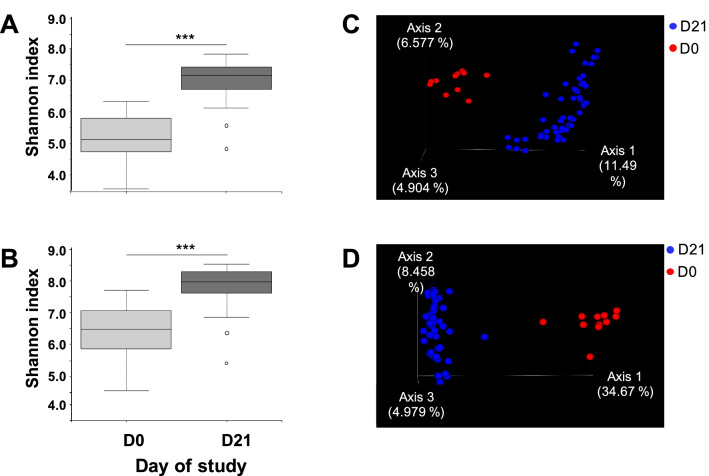
Alpha and beta diversity of faecal and nasal microbiota change through time. Alpha diversity estimated using Shannon’s index on rarefied samples from fecal content (**A**) or nasal swabs (**B**) compared before starting the treatment with feed addtives (D0) and after 21 days of treatment (D21). The dotted lines represent standard deviation and outliers are indicated with white circles. Beta diversity analysis on Bray Curtis’ distances of the faecal (**C**) and nasal (**D**) microbiota composition among samples collected at D0 and D21. The principal axis are shown with the percentage of variation explained between brackets. *** stands for *P* value < 0.001.

Alpha diversity, measured by the Shannon index, of the faecal microbiota increased from D0 to D21 (Fig. 2A; Shannon, *P* = 0.000001), together with the richness (Chao, *P* = 0.00002). The same trend was observed in the nasal microbiota, where alpha diversity (Fig. 2B; Shannon, *P* = 0.000006) and richness (Chao, *P* = 0.017) also increased significantly during the study.

Beta diversity analysis showed a clear clustering of the faecal microbiota composition on D0 and D21, with the quantitative analysis explaining better the differences between the composition at these two timepoints (Bray Curtis R^2^ = 10.10%, Fig. 2C; Jaccard R^2^ = 8.58%; *P* = 0.001). Regarding the nasal microbiota, beta diversity analysis showed differential clustering by both Bray–Curtis and Jaccard indexes, where the difference explained by this grouping was also higher in the quantitative approach (Bray Curtis R^2^ = 33.50%, Fig. 2D; Jaccard R^2^ = 12.54%; *P* = 0.001). In order to detect differences in microbial mean taxa abundance over time, we performed the analysis of composition of microbiomes (ANCOM) that is based in the assumption that inter-taxa ratios are maintained for non-differentially abundant taxa. ANCOM performed at genus level detected 20 genera in faeces and 37 in nasal microbiota composition that dynamically changed over time (Supplementary Table S1).

### Alpha diversity of faecal and nasal microbiota composition after treatments

**Figure 3:**
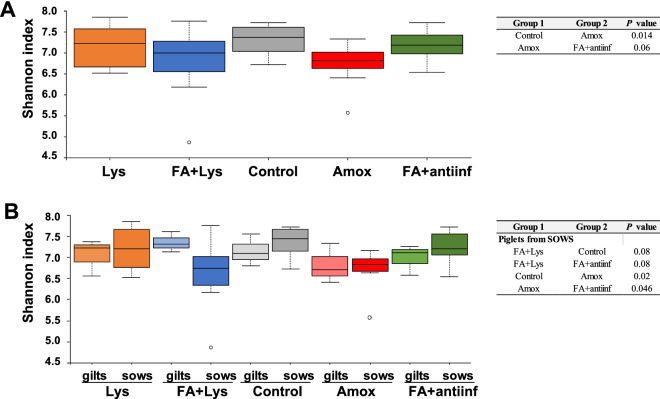
Alpha diversity of faecal microbiota is different depending on the treatment group. Diversity was estimated using Shannon’s index at D21 on faecal samples for the treatment groups (**A**) or considering the parity of the dams (**B**). The dotted lines represent standard deviation and outliers are indicated with white circles. The alpha significance is shown in the right panel indicating the *P* values, when significant. The treatment groups were: *Lys* lysozyme, *FA + lys* medium chain fatty acids and lysozyme, *C* control, with no additives, *Amox* amoxicillin, *FA + antiinf* medium chain fatty acids and a natural antiinflammatory; each group was splitted according the parity of their dams: gilts (first delivery) or sows (more than 1 delivery).

Differences in diversity among treatments were explored by comparing the microbiota composition after 3 weeks of treatment (D21). Alpha diversity of the faecal microbiota was significantly lower in the Amox group when compared to the control group (Fig. 3A, P = 0.01) and demonstrated a tendency of being lower than the diversity found in the group FA + antiinf (*P* = 0.06). When the parity of the dams (gilts vs sows) was considered in the analysis, piglets born to sows from the Amox group showed lower diversity than pigs from control and FA + antiinf groups born to sows (Fig. 3B). Alpha diversity from the piglets born to sows from the FA + Lys group showed a tendency to be lower than the diversity observed in the piglets born to sows in the control and FA + antiinf groups (*P* = 0.08 in both cases; Fig. 3B). Although some differences in Shannon index were observed in the piglets born to gilts, these differences were not statistically significant under any of the treatments. In addition, the richness of the faecal microbiota in the Amox group was lower than the control group (Chao, *P* = 0.016) but showed a trend to be higher than the Lys group (Chao, *P* = 0.08). When explored considering the parity of the dams (Supplementary Figure S1A), the lower richness in the Amox group compared to control group was only confirmed in animals born to gilts (*P* = 0.049). Also, FA + antiinf group showed lower richness than the control group (*P* = 0.05) considering only animals born to gilts. However, considering only animals born to sows other difference in richness became evident, i.e. Lys + FA group showed lower richness compared to Lys group (*P* = 0.047, Supplementary Figure S1A). In general, pigs born to gilts had lower richness in the faecal microbiota than those born to sows (*P* = 0.037, Supplementary Figure S1B).

**Figure 4:**
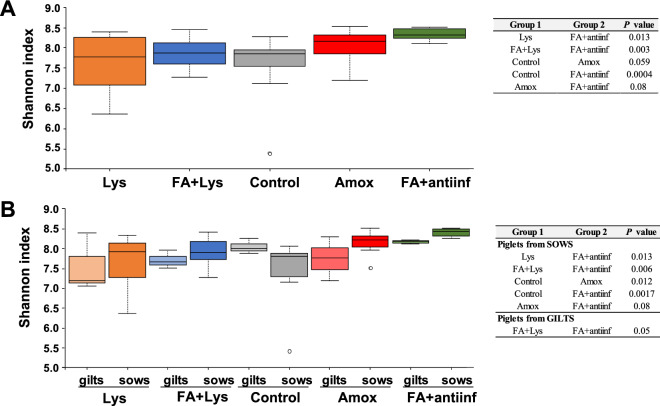
Alpha diversity from nasal microbiota is different depending on the treatment group. Diversity was estimated using Shannon’s index at D21 on nasal samples for the treatment groups (**A**) or considering the parity of the dams (**B**). The dotted lines represent standard deviation and outliers are indicated with white circles. The alpha significance is shown in the right panel indicating the *P* values, when significant. The treatment groups were: *Lys* lysozyme, *FA + lys* medium chain fatty acids and lysozyme, *C* control, with no additives, *Amox* amoxicillin, *FA + antiinf* medium chain fatty acids and a natural anti-inflammatory; each group was splitted according the parity of their dams: gilts (first delivery) or sows (more than 1 delivery).

Alpha diversity of the nasal microbiota was also compared among the treatment groups on D21 (Fig. 4A). The FA + antiinf group showed the highest diversity when compared against all the other groups, including the control (*P* = 0.0004), although it was only a tendency when compared to the Amox group (*P* = 0.08). In contrast to what was observed in the faecal microbiota, the diversity of the group under antibiotic treatment (Amox) tended to be higher than the control group (*P* = 0.06). The analysis separating the piglets depending on the parity of the dams, revealed that this factor also affected the nasal composition (Fig. 4B). The nasal microbiota from piglets born to sows showed similar results than those obtained considering all the animals together, with the FA + antiinf group showing the highest diversity among all the treatments. The antibiotic treatment increased the diversity in the nasal microbiota in pigs born to sows (piglets from sows: Amox vs Control, *P* = 0.012) but it did not affect the diversity in pigs born to gilts (piglets from gilts: Amox vs control, *P* = 0.51). In general, the nasal diversity in piglets born to gilts was mildly affected by the treatment. Alpha diversity was higher in piglets born to gilts in the control and FA + antiinf groups, but no group showed significant differences when compared with the control group. However, alpha diversity in the nasal microbiota of piglets born to gilts from the FA + Lys group was significantly lower than their counterparts from the FA + antiinf group. Noteworthy, the comparison between piglets born to sows or gilts within treatments showed a different trend in the control group than in the rest of treatments (Fig. 4B). Control piglets from sows showed lower diversity than those from gilts (*P* = 0.09), while in the rest of treatments piglets from sows showed higher diversity than those from gilts, although this difference was only significant in the FA + antiinf group (*P* = 0.017).

In agreement with the higher alpha diversity found by Shannon index, groups FA + antiinf (FA + antiinf vs Lys *P* = 0.07; FA + antiinf vs FA + Lys *P* = 0.03; FA + antiinf vs control *P* = 0.07) and Amox (Amox vs Lys *P* = 0.01; Amox vs FA + lys *P* = 0.01; Amox vs control *P* = 0.016) showed the highest species richness in the nasal microbiota, with no differences between them. Considering the parity of the dams, we found the same trends when comparing only animals born to sows (i.e. both FA + antiinf and Amox groups showing the highest richness, Supplementary Figure S1C), while some of these differences were lost when considering only animals born to gilts only. In general, the parity of the dams affected the richness of the nasal microbiota composition of their offspring in the same way it affected the faecal microbiota, since animals born to gilts showed the lowest richness independently of the treatment group they belonged (gilts vs sows, *P* = 0.021; Supplementary Figure S1D).

### Beta diversity of faecal and nasal microbiota composition after treatments

The PCoA of the faecal microbiota on D21 revealed that approximately 9% of the differences in microbiota composition could be explained by the treatment in both, quantitative and qualitative analyses (Bray Curtis R^2^ = 9.50% *P* = 0.05; Jaccard R^2^ = 9.21%, *P* = 0.01; Supplementary Figure S2). The pairwise PERMANOVA analysis on Bray Curtis distances revealed differences between some of the treatment groups, specially for Amox group (Lys vs Control, *P* = 0.06; Amox vs Lys + FA, *P* = 0.08; Amox vs Control, *P* = 0.08). When parity (gilts vs sows) was included in the analysis, the PCoA demonstrated that clustering by treatment was significant when considering the qualitative approach of the Jaccard index (R^2^ = 19.61%, *P* = 0.036), but failed in a quantitative approach (Bray Curtis R^2^ = 19.75%, *P* = 0.127). Interestingly, differences by treatment could be traced back mainly to the piglets born to gilts, since PCoA on these animals was significative in quantitative approach explaining a higher percentage of the differences (Bray Curtis R^2^ = 40.25%, *P* = 0.05; Jaccard R^2^ = 38.04%, *P* = 0.08). For the piglets born to sows, clustering was not explained by treatment using either qualitative or quantitative approaches (Jaccard *P* = 0.6, Bray Curtis *P* = 0.2).

The PCoA on the nasal microbiota composition showed that the FA + antiinf group was more homogenous, i.e. piglets in this group had a more similar microbiota composition among them, in contrast with the other groups (Supplementary Figure S3). When the clustering was analysed based on treatment group, the differences were better explained through quantitative (Bray Curtis R^2^ = 23.71%, *P* = 0.001) than qualitative analysis (Jaccard R^2^ = 15.04%, *P* = 0.001). When the clustering analysis was performed considering both the treatments and the parity of the dams (sows *versus* gilts) the differences were better explained (Bray Curtis R^2^ = 33.26%, *P* = 0.001; Jaccard R^2^ = 25.883%, *P* = 0.001), indicating that the parity of the dams had also an effect on the nasal microbiota. The pairwise PERMANOVA analysis over Bray Curtis distances showed that all the comparisons were statistically significant among all treatment groups (*P* < 0.01). Clustering by treatment was significatively explained in piglets from sows (Bray Curtis R^2^ = 29.74%, *P* = 0.001; Jaccard R^2^ = 18.84%, *P* = 0.001), as well as in piglets from gilts (Bray Curtis R^2^ = 42.85%, *P* = 0.001; Jaccard R^2^ = 36.97%, *P* = 0.001).

**Figure 5:**
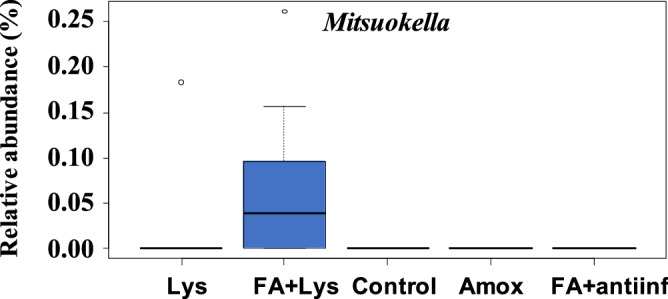
Analysis of composition of microbiomes (ANCOM) on fecal microbiota at genus level comparing groups after treatment (D21). *Mitsuokella* genus was dected with this differential abundance test. The mean relative abundance of this genus is shown in percentage. Each boxplot corresponds to one treatment group: *Lys* lysozyme, *FA + lys* medium chain fatty acids and lysozyme, *C* control, with no additives, *Amox* amoxicillin, *FA + antiinf* medium chain fatty acids and a natural anti-inflammatory. The dotted lines represent standard deviation and outliers are indicated with white circles.

**Figure 6:**
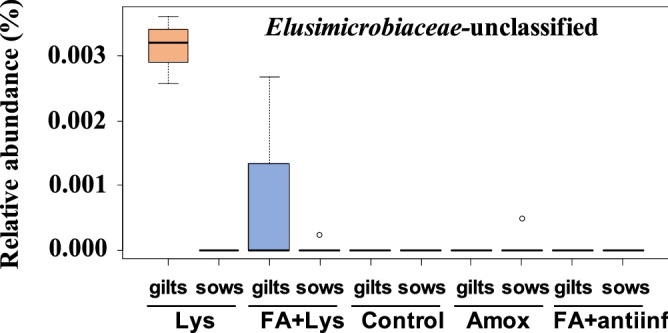
Analysis of composition of microbiomes (ANCOM) on fecal microbiota comparing groups considering the parity of the dams after treatment (D21). The ANCOM at amplicon sequence (ASV) level detected an unclassified genus from *Elusimicrobiacea* family with differential abundance when compared groups considering the parity of their dams. The mean relative abundance of this ASV is shown in percentage. The dotted lines represent standard deviation and outliers are indicated with white circles. The treatment groups were: *Lys* lysozyme, *FA + lys* medium chain fatty acids and lysozyme, *C* control, with no additives, *Amox* amoxicillin, *FA + antiinf* medium chain fatty acids and a natural antiinflammatory; each group was splitted according the parity of their dams: gilts (first delivery) or sows (more than 1 delivery).

ANCOM was performed on samples from D21 to detect differentially abundant genera among treatments. When the faecal microbiota was compared across all the samples, only one genus, *Mitsuokella* was identified (Fig. 5). However, no genus was detected when the parity of the dams was included in the analysis. In addition, ANCOM was performed to detect differential abundant ASVs in the faecal microbiota under different treatments, but none was detected. When the parity of the dams was considered, one ASV classified in the *Elusimicrobiaceae* family was detected to be differentially abundant among the treatments (Fig. 6). This ASV was only detected in the faecal microbiota of the piglets from gilts in the Lys and FA + Lys treatments, although in very low abundance.

**Figure 7:**
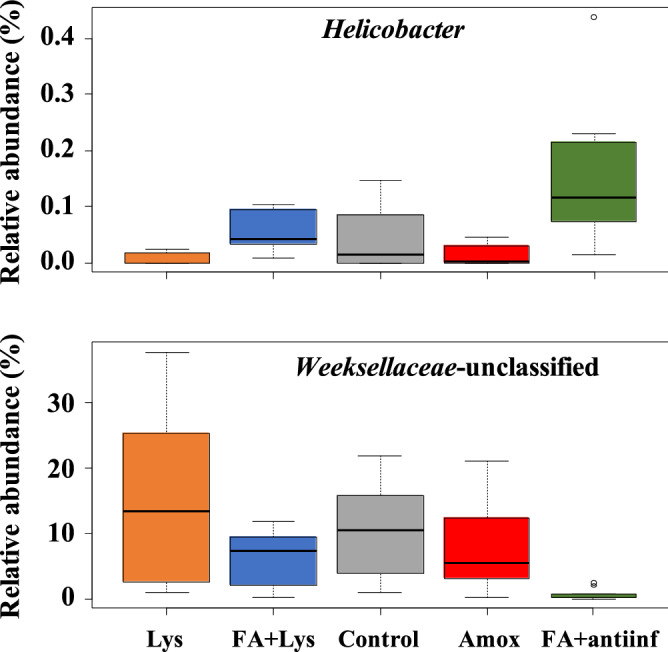
Analysis of composition of microbiomes (ANCOM) on fecal microbiota at genus level comparing groups after treatment (D21). *Helicobacter* genus and unclassified member from *Weeksellaceae* family were dected with this differential abundance test. The mean relative abundance of these genera is shown in percentage. Each boxplot corresponds to one treatment group: *Lys* lysozyme, *FA + lys* medium chain fatty acids and lysozyme, *C* control, with no additives, *Amox* amoxicillin, *FA + antiinf* medium chain fatty acids and a natural anti-inflammatory. The dotted lines represent standard deviation and outliers are indicated with white circles.

**Figure 8:**
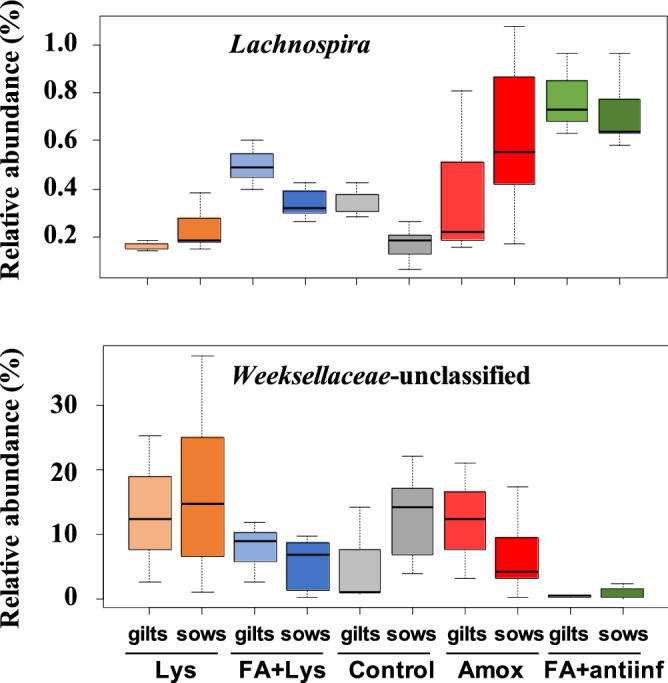
Analysis of composition of microbiomes (ANCOM) on nasal microbiota comparing groups considering the parity of the dams after treatment (D21). The ANCOM at genus level detected *Lachnospira* and an unclassified member from *Weeksellaceae* as differentially abundant. The mean relative abundance of these genera is shown in percentage. The dotted lines represent standard deviation and outliers are indicated with white circles. The treatment groups were: *Lys* lysozyme, *FA + lys* medium chain fatty acids and lysozyme, *C* control, with no additives, *Amox* amoxicillin, *FA + antiinf* medium chain fatty acids and a natural antiinflammatory; each group was splitted according the parity of their dams: gilts (first delivery) or sows (more than 1 delivery).

**Figure 9:**
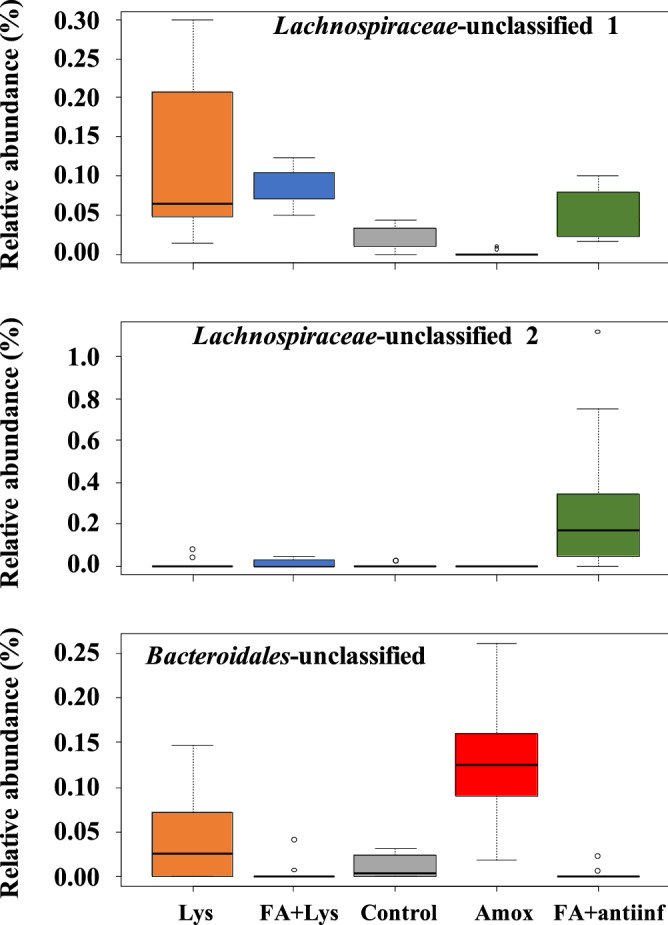
Analysis of composition of microbiomes (ANCOM) on nasal microbiota comparing groups after treatment (D21). The ANCOM at amplicon sequence variant (ASV) level detected two unclassified members from *Lachnospiraceae* family and one member from *Bacteroidales* order as differentially abundant. The mean relative abundance of these ASVs is shown in percentage. Each boxplot corresponds to one treatment group: *Lys* lysozyme, *FA + lys* medium chain fatty acids and lysozyme, *C* control, with no additives, *Amox* amoxicillin, *FA + antiinf* medium chain fatty acids and a natural antiinflammatory. The dotted lines represent standard deviation and outliers are indicated with white circles.

**Figure 10:**
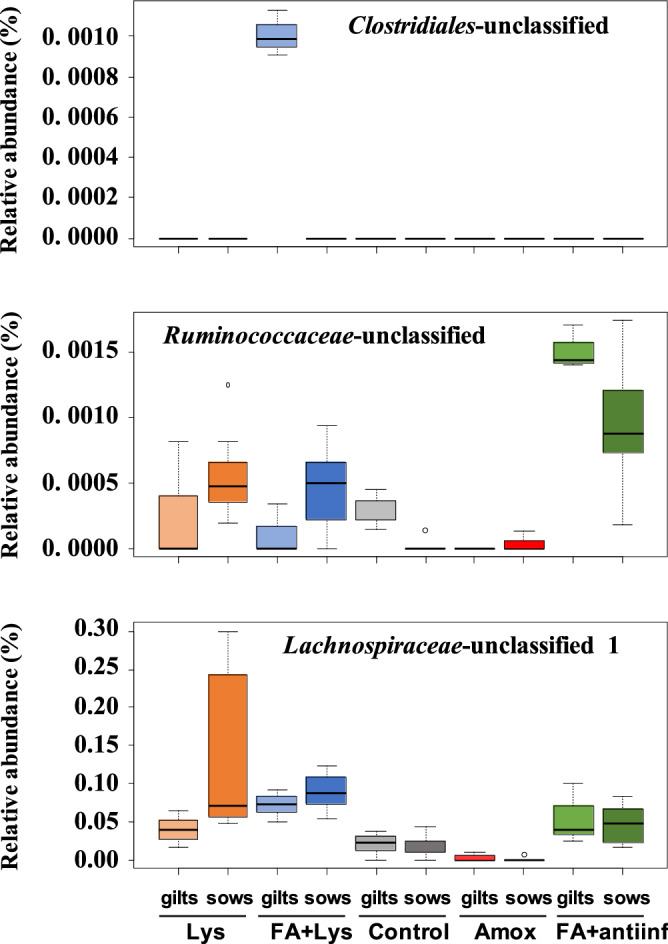
Analysis of composition of microbiomes (ANCOM) on nasal microbiota comparing groups considering the parity of the dams after treatment (D21). The ANCOM at amplicon sequence variant (ASV) level detected two unclassified members, one from *Lachnospiraceae* and other from *Ruminococcaceae* family, and one member from *Clostridiales* order as differentially abundant. The mean relative abundance of these ASVs is shown in percentage. The dotted lines represent standard deviation and outliers are indicated with white circles. The treatment groups were: *Lys* lysozyme, *FA + lys* medium chain fatty acids and lysozyme, *C* control, with no additives, *Amox* amoxicillin, *FA + antiinf* medium chain fatty acids and a natural antiinflammatory; each group was splitted according the parity of their dams: gilts (first delivery) or sows (more than 1 delivery).

In the nasal microbiota, ANCOM detected that *Helicobacter* and an unclassified genus from the *Weeksellaceae* family were differentially abundant among the treatments (Fig. 7). When parity was included in the analysis, *Lachnospira* and an unclassified genus from *Weeksellaceae* were detected (Fig. 8). ANCOM was also performed to detect differential ASVs from the nasal microbiota and identified 2 ASVs from *Lachnospiraceae* and 1 ASV from *Bacteroidales* (Fig. 9). When parity was included in the analysis, 3 ASVs were detected: 1 from *Clostridiales*, 1 from *Lachnospiraceae* (one of the two detected by treatment only) and 1 from *Ruminococcaceae* (Fig. 10).

## Discussion

Antimicrobial metaphylaxis is still used as part of control programmes for respiratory diseases in conventional pig farming, and the treatment with broad spectrum beta-lactam antimicrobials is considered the most effective against *S. suis* infections^5^. Efforts are being carried out by the swine sector to reduce the use of antimicrobials, but controlling the transmission of *S. suis* still represents a challenge, since this pathogen is widely distributed in the majority of pig farms^5^. Accordingly, our study confirmed the presence of *S. suis* in nearly all piglets sampled at weaning^11^. None of the treatments, including the antimicrobial, reduced the prevalence of colonization by *S. suis*, or by other potential pathogens, such as *G. parasuis*.

The productive performance of the piglets (ADWG) did not appear to be affected by any of the treatments. However, it is important to notice that diseased animals were excluded from the analysis of this parameter and, particularly, the control and the FA + antiinf groups had the highest number of animals removed (39 and 30, respectively). The ADWG values in these two groups were widely scattered, with large differences among animals. In contrast, ADWG was less dispersed in the Amox group. This farm was under the infection pressure of *S. suis*, and the effect of these additives on ADWG in a farm with a good health status would need to be evaluated to reach appropriate conclusions on this parameter. Additionally, animals were not followed until the end of the rearing cycle, and therefore we cannot do any inference on the effect of the treatments at the end of the fattening period, which is the time when this value is more meaningful.

Since there is a call for reducing the use of antimicrobials in veterinary medicine, additives could be a potential alternative to the use of amoxicillin, which is known to favour emergence of resistant bacteria. Clinical signs compatible with *S. suis* obtained supplementing the food with medium chain fatty acids combined with a natural anti-inflammatory (FA + antiinf) were at the same level than those obtained with amoxicillin. High alpha diversity in the microbiota from different body sites, including the nose, was shown to be associated with good health in both, animal and human hosts^12–15^. Nasal microbiota is probably important in the first steps of *S. suis* infection since the upper respiratory tract is a natural entry route for this pathogen^16^. Accordingly, the group with the highest diversity of microbial communities in the nasal cavities was group FA + antiinf, which in turn showed the lowest *S. suis* disease prevalence. Regarding general health status (in this particular farm, clinical signs included limping and nervous signs, wasting, diarrhea and coughing), the results obtained in two treatment groups, Lys and FA + lys, were similar to the group treated with amoxicillin, confirming, once again, that the use of amoxicillin could be reduced. Unexpectedly, the nasal diversity was higher in the Amox group compared to the control. Since the nose is more exposed to environmental changes, the increased number of species detected could be a result of transient bacteria that are not actual colonizers, and do not find much competition in a microbiota depleted by the antimicrobial treatment. However, this effect was not observed in previous studies^17–19^ and may depend on several factors, including the age of the animals, the type of antimicrobial or the duration of treatment. Hence, this intriguing finding deservers further investigation to understand this phenomenon in the nasal microbiota.

It is well established that the microbiota of the piglets evolves rapidly during the first weeks of life, increasing the diversity and community richness of bacterial species^20,21^, as it was confirmed herein for faecal and nasal microbiota. Animal health and production during different growth stages have been correlated with components of the swine gut microbiome in several studies^22–24^. At weaning, piglets’ diet changes from milk to dry food, and this has an impact in the bacterial communities of the microbiota^25,26^. Among the major changes detected in the faecal microbiota composition along time, *Prevotella* and *Bacteroides* genera showed an opposite behaviour, increasing from a mean of 1.25–10.5% and decreasing from 18.3 to 0.52% respectively, in agreement to what was previously reported by Frese et al.^22^. The relative increased in *Prevotella* abundance may be essential for plant-based digestion in post-weaning piglets^32^. We also detected a decreasing tendency in *Lactobacillus* throughout time that might be also related to the changes in feeding^27^, since members from this genus are early colonizers that depend on milk’s lactose, which are normally replaced by other genera later in time^28^. Regarding major changes in nasal microbial composition after weaning, we found the same *Bacteroides* and *Prevotella* inverse apparent association observed in the faeces. Remarkably, several genera associated to health (such as *Lachnospira* and *Blautia* from *Lachnospiraceae,* and *Faecalibacterium* and *Ruminococcus* from *Ruminococcaceae*) relatively increased, probably promoting the immune and anti-stress functions^29^, while *Bergeyella* and *Fusobacterium*, which have been associated to disease^30–32^, showed to relatively decrease over time.

To our surprise, diet supplements did not have a major effect on the composition of the faecal microbiota, while the effect was more evident on the nasal microbiota composition. The only genus differentially present in the faecal microbiota when compared treatments, was *Mitsuokella,* which was only detected in FA + Lys group. This genus was associated to *Prevotella* as an important co-occurrent genus to define enterotype-like clustering structure of the faecal microbiota in pigs^33^, revealing the importance of this genus. On the other hand, the analysis perform at ASV level showed one ASV from *Elusimicrobiaceae* family, which was only detected in the piglets born to gilts from groups Lys and Lys + FA in very low abundance. Noteworthy, members from this family have been negatively associated to backfat accumulation, average daily gain and residual feed intake in a study where feeding behaviour traits were under analysis in pigs^34^. However, we did not observe differences in average daily weight gained when analysing this parameter by parity of the dams.

In the nasal microbiota, several genera were differentially abundant when treatments were compared, including members of *Weeksellaceae* family. This family is found in high abundance in the nasal cavities of healthy piglets, although in increased relatively abundance in the core microbiota of animals with respiratory disease^32,35^. Interestingly, this particular unclassified member from this family was in lower abundance in the nasal microbiota of pigs from the FA + antiinf group, which developed the lowest percentage of clinical signs compatible with *S. suis* disease. In contrast, the analysis at ASV level found one ASVs from *Lachnospiraceae* that was more relatively abundant in the FA + antiinf group. This opposite occurrence between these two taxa deserves further investigation to unveil the potential implications in animal health.

The distinctive clustering of both, faecal and nasal samples was explained by combining treatment and dam parity better than by just treatment. This analysis unveiled that parity of the dams impacted the development of the early microbiota. To the best of our knowledge, no other reports in the literature have described the effect of parity on the microbial composition of their offspring. However, several studies have reported increasing cases of diarrhea in litters born to gilts^36,37^, which could be a consequence of a lower microbiota diversity. In general, we found that piglets born to gilts contained less microbial diversity in all treatment groups in the nasal and faecal microbiota, perhaps as a consequence of the antimicrobial treatment performed to gilts for aclimatation to the farm. Other factors can not be ruled out, suh as prenatal stress or milk quality. Prenatal stress has been demonstrated to have an impact in microbial colonization in new-born babies^38^, and this stress could be higher in gilts than in sows^39^. Lactation has been shown to impact the neonatal microbiota, not only because of the abundant bacterial communities in milk, but also because milk is a rich and natural source of oligosaccharides that may have prebiotic activity^40^. It is not known if the composition of the milk microbiota differs depending the parity of the dam, however this usually correlates with age, and the microbial communities change along time in many body sites^41^. The development of the microbiota during early life sets the stage for the adult microbiome and can have long-term impacts on the health of the host^42,43^. Therefore, specific interventions directed to gilts aiming at reducing stress and improve nutritional programmes by adding prebiotics or probiotics during gestation to promote the growth of beneficial bacteria would have a positive effect in the litter.

In conclusion, the use of amoxicillin resulted in a consistent reduction of clinical signs, but the evaluated additives have been shown to be a good alternative in the present scenario of reduction of antimicrobials. The feed supplementation with medium chain fatty acid and lysozyme improved clinical signs for general disease (coughing, diarrhea and wasting), whereas supplementation with medium chain fatty acids and a natural antiinflamatory reduced clinical signs compatible with *S. suis*. Interestingly, the effect of the feed additives was more significant in the nasal microbiota than in the faecal microbiota despite the oral administration of these. Moreover, sow parity influenced the microbiota composition in both faeces and nose. Taking together, these results highlight the importance of understading the dynamics of the microbiota, and the complexity of formulating nutritional programs to manipulate the microbiota composition toward the right balance to improve health and prevent diseases.

## Materials and methods

### Study design

A farm with recurrent problems of disease confirmed to be caused by *S. suis* was selected for the study. Sampling of piglets was done under institutional authorization and followed good veterinary practices. According to European (Directive 2010/63/EU of the European Parliament and of the Council of 22 September 2010 on the protection of animals used for scientific purposes) and Spanish (Real Decreto 53/2013) normatives, this procedure did not require specific approval by an Ethical Committee. Faecal and nasal sampling is not likely to cause pain, suffering, distress or lasting harm equivalent to, or higher than, that caused by the introduction of a needle in accordance with good veterinary practice (Chapter I, Article 1, 5 (f) of 2010/63/EU). All experiments were performed in accordance with relevant guidelines and regulations.

A whole batch of new-born piglets from 54 dams were designated to take part in the study. Dams included did not take any medication 15 days prior farrowing or during farrowing. One-day-old piglets were ear-tagged and the routine procedure established by the veterinarian of the farm in terms of vaccination (circovirus vaccine at 3 weeks of age) was carried out.

One week before weaning, all piglets (n = 569) were ear-tagged on the second ear and animals were weighed. Piglets were randomly distributed into five experimental groups by dam’s origin (balanced proportion of piglets of each dam in each of the experimental group) and weight (same average weight per group). For analysis of *S. suis* and *Glaesserella* (*Haemophilus*) *parasuis* nasal colonization in the different treatment groups, a subset of 125 piglets were randomly selected representing one piglet per litter in each of the treatment-groups (25 piglets per treatment). Cross-fostered piglets were not included in this selection. Nasal samples were taken in this subset of animals at weaning (D0) and after 3 weeks of treatment (D21). Additionally, nasal and faecal samples were taken from a subset of 12 piglets at D0 and 10 piglets per group at D21 for microbiota analysis.

Twenty-one days-old piglets were weaned and moved from the farrowing unit and allocated into 5 groups. Five different treatments (two treatments per room) were assigned; treatments Lys (feed supplemented with lysozyme, both free and encapsulated, n = 113), FA + Lys (feed supplemented with medium chain fatty acids (FA) and lysozyme, n = 110), Amox (in-feed amoxicillin, n = 115), and FA + antiinf (feed supplemented with Naturporc, containing medium chain FA and a natural anti-inflammatory, n = 119). Also a control group of piglets was included (control feed with no additives, n = 112).

Animals were followed up daily by the personnel at the farm; however, a closer clinical evaluation was performed every two days by the veterinarian until departure to the fattening farm. Recording of clinical signs was carried out individually. Based on the veterinarian assessment, animals that needed antibiotic treatment during the course of the study were removed and the cause of removal was annotated. Three animals were euthanized and sampled during the study period, two of them presenting clinical signs compatible with the presence of *S. suis*, which were limping and nervous signs. Animals that died during the study were also recorded and the presumptive cause of dead was annotated in the database. To avoid subjected interpretation of results, the veterinarian and personnel at the farm were unaware of the additives used in each of the treatment groups. All animals in the five groups (n = 434) were weighed at the end of the study period (day 43).

### Isolation and identification of *S. suis* and *G. parauis*

Individual nasal swabs were taken in two different occasions from the subset of 125 pigs and kept at -80ºC until analysis at the end of the study. Nasal swabs were resuspended in PBS and DNA was extracted using a NucleoSpin Blood kit (Macherey–Nagel, Germany) following manufacturer´s recommendations. DNA was tested for the presence of *S. suis* and *G. parasuis* with primers and conditions described before^44,45^. PCR for *G. parasuis* has been designed to detect virulence-associated trimeric autotransporters (*vtaA*) genes associated to *G. parasuis* virulent strains^45^. Although *S. suis* was suspected to be the cause of disease in this specific farm, *G. parasuis* was included in the diagnosis since the clinical signs of both infections are similar and both pathogens affect piglets of the same age, mainly piglets in the nursery phase.

Swabs obtained from the foramen magnum of sacrificed pigs with clinical signs compatible with infection by *S. suis* were plated in chocolate agar (Biomerieux, Spain) and incubated overnight at 37 C with 5% CO_2_. Once the presence of *S. suis* was confirmed, identification of serotypes 2 and 9 was assessed as described elsewhere^46^.

*S. suis* isolated strains were genotyped by ERIC-PCR in order to assess the presence of different disease-causing strains on the farm, as was described by Versalovic et al*.*^47^.

### Antimicrobial susceptibility testing

Antimicrobial susceptibility testing was performed by broth microdilution methods using commercial plates (Sensititre) and following guidelines described by the Clinical and Laboratory Standards Institute (CLSI). Antimicrobials tested were chlortetracycline (0.5–8 mg/L), oxytetracycline (0.5–8 mg/L), florphenicol (0.25–8 mg/L), penicillin (0.12–8 mg/L), ampicillin (0.25–16 mg/L), ceftiofur (0.25–8 mg/L), gentamicin (1–16 mg/L), neomycin (4–32 mg/L), spectinomycin (8–64 mg/L), tiamulin (1–32 mg/L), sulphadimethoxine (256 mg/L), trimethoprim/sulfamethoxazole (2/38 mg/L), tylosine (0.5–32 mg/L), tulathromycin (1–4 mg/L), tilmicosin (4–64 mg/L), clindamycin (0.25–16 mg/L), danofloxacin (0.12–1 mg/L) and enrofloxacin (0.12–2 mg/L). Clinical breakpoints were described by CLSI (CLSI, 2020).

### Statistical analyses

The prevalences of disease compatible with *S. suis* infection (based on clinical signs) in the different treatment groups first compared with the prevalences of disease attributed to *S. suis* in both, the group treated with amoxicillin and the negative control group. Comparisons were carried out using the Pearson's Chi-squared test.

Similar comparisons were carried out but using the total prevalence of disease in the different treatment groups. The total prevalence included not only disease caused by *S. suis*, but also wasting, diarrhea and/or coughing. Comparisons were also carried out using the Pearson's Chi-squared test.

Finally, the average daily weight gain in the five treatment groups were also compared. In order to do that, the normality of data in the different groups was assessed using the Shapiro–Wilk test. In case of normally distributed data, the weights were compared with an Anova test, with a post-hoc Tukey test to adjust for multiple comparisons when the Anova test was statistically significant. In case of non-normally distributed data, the weights in the different groups were compared with a Kruskal–Wallis test, with a post-hoc Dunn's test with Bonferroni correction to adjust for multiple tests when the Kruskal–Wallis test was statistically significant. The significance level (*P* value) was set at 0.05. All the statistical analyses were carried out using R statistical software (https://cran.r-project.org/).

### Microbiota analyses

A subset of 10 clinically healthy individuals per treatment group representing litters from 10 different dams and proportionally balanced in the five treatment groups were selected for nasal and faecal microbiota analyses. Nasal swabs and faecal contents were obtained after three weeks to assess the effect of the different treatments on the microbiota. As control, nasal and faecal samples from 12 piglets obtained at weaning (D0), representing the microbiota composition before the treatments started, were also included in the analysis. Bacterial DNA was extracted from 0.2 g of homogenised faeces using a NucleoMag VET kit (Cultek) following the manufacturer’s instructions. DNA from nasal swabs was extracted as stated above with a NucleoSpin Blood kit (Macherey–Nagel, Germany). Quality and quantity of DNA were evaluated on a BioDrop DUO (BioDrop Ltd, Cambridge. UK). The library preparation for sequencing was performed at The Carver Biotechnology Center (University of Illinois, United States) for rectal samples and at *Servei de Genòmica* (*Universitat Autònoma de Barcelona*) for nasal samples. In either case, sequencing of 16S rRNA gene was done with Illumina MiSeq pair-end 2 × 250 bp technology following the manufacturer instructions (MS-102-2003 MiSeq Reagent Kit v2, 500 cycle). The region targeted to perform the 16S amplification was the one spanning the V3 and V4 region of 16S rRNA gene selected from Klindworth et al*.*^48^.

For the taxonomic analysis, both Qiime2 v2019.10^49^ and in-house scripts were used. Raw reads were evaluated and quality-filtered with q2-demux plugin. Trimming and denoising with DADA2^50^ allowed the identification of amplicon sequence variants (ASVs). ASVs were aligned using MAFFT^51^ and a phylogenetic tree was built using Fasstree2^52^. Taxonomic assignment was performed with q2‐feature‐classifier^53^ classify‐sklearn naïve Bayes taxonomy classifier against the Greengenes (version 13.8) database^54^.

Alpha diversity indexes (Shannon, Chao) were calculated on rarefied 16S rRNA gene sequence data at maximum depth for all samples (which was 8500 from rectal samples and 19,000 for nasal samples). Alpha diversity between groups was compared through two-sample non-parametric t-tests (Monte Carlo method) at maximum depth in rarefied samples (with 999 permutations). In addition, equal number of samples was subsampled to assess the significant differences between sample types. The plugin q2-diversity core-metrics was used to both, estimate beta diversity metrics and perform Principal Coordinate Analysis (PCoA). We used different metrics to represent the differences in community composition, where Jaccard reports differences in the presence or absence of ASVs, Bray–Curtis accounts for these differences but also includes abundance of the ASVs. PCoA were visualized using EMPeror^55^. The percentage of variation between grouped samples was measured by R2, using Adonis function of the vegan package in R software^56^. Estimation of *P* values was done through Monte Carlo test with 999 random permutations of the data set. Permutational analysis of the variance (PERMANOVA) was performed to compare beta diversity matrices over the treatments under study with 999 permutations^57^ (*q2 diversity beta-group-significance*). In order to identify differentially abundant taxa between treatment groups, the test of analysis of composition in microbiomes (ANCOM) was used on the tables previously filtered by frequency (n > 10) and collapsed by genus (L6). Boxplots were built using boxplots function^58–60^ in R^61^. In all cases, samples were considered to be significantly different when the accompanying *P value* was ≤ 0.05.

## Supplementary information

Supplementary Information

## Data Availability

The entire sequence dataset is available in the Sequence Read Archive (SRA) database from NCBI, BioProject PRJNA632930 and BioSamples SAMN14928473-14928596.

## References

[1] Davies J, Davies D (2010). Origins and evolution of antibiotic resistance. Microbiol. Mol. Biol. Rev..

[2] Seitz M, Valentin-Weigand P, Willenborg J (2016). Use of antibiotics and antimicrobial resistance in veterinary medicine as exemplified by the Swine Pathogen *Streptococcus suis*. Curr. Top. Microbiol. Immunol..

[3] Callens B, Persoons D, Maes D, Laanen M, Postma M, Boyen F (2012). Prophylactic and metaphylactic antimicrobial use in Belgian fattening pig herds. Prev. Vet. Med..

[4] Cloutier G, D'Allaire S, Martinez G, Surprenant C, Lacouture S, Gottschalk M (2003). Epidemiology of *Streptococcus suis* serotype 5 infection in a pig herd with and without clinical disease. Vet. Microbiol..

[5] Hopkins D, Poljak Z, Farzan A, Friendship R (2018). Factors contributing to mortality during a *Streptoccocus suis* outbreak in nursery pigs. Can. Vet. J..

[6] Sanford SE, Tilker ME (1982). *Streptococcus suis* type II-associated diseases in swine: observations of a one-year study. J. Am. Vet. Med. Assoc..

[7] Varela NP, Gadbois P, Thibault C, Gottschalk M, Dick P, Wilson J (2013). Antimicrobial resistance and prudent drug use for *Streptococcus suis*. Anim. Health Res. Rev..

[8] Communication from the Commission to the European Parliament and the Council ‐ Action plan against the rising threats from Antimicrobial Resistance, in *COM (2011) 748. AMR Road map ‐ Action no 10.*, E. Commission, Editor. Brussels, Belgium (2011).

[9] Heo JM, Opapeju FO, Pluske JR, Kim JC, Hampson DJ, Nyachoti CM (2013). Gastrointestinal health and function in weaned pigs: a review of feeding strategies to control post-weaning diarrhoea without using in-feed antimicrobial compounds. J. Anim. Physiol. Anim. Nutr. (Berl).

[10] Lalles JP, Bosi P, Smidt H, Stokes CR (2007). Nutritional management of gut health in pigs around weaning. Proc. Nutr. Soc..

[11] MacInnes JI, Gottschalk M, Lone AG, Metcalf DS, Ojha S, Rosendal T (2008). Prevalence of *Actinobacillus pleuropneumoniae*, *Actinobacillus suis*, *Haemophilus parasuis*, *Pasteurella multocida*, and *Streptococcus suis* in representative Ontario swine herds. Can. J. Vet. Res..

[12] Megahed A, Zeineldin M, Evans K, Maradiaga N, Blair B, Aldridge B (2019). Impacts of environmental complexity on respiratory and gut microbiome community structure and diversity in growing pigs. Sci. Rep..

[13] Gresse R, Chaucheyras Durand F, Duniere L, Blanquet-Diot S, Forano E (2019). Microbiota composition and functional profiling throughout the gastrointestinal tract of commercial weaning piglets. Microorganisms.

[14] Larsen OFA, Claassen E (2018). The mechanistic link between health and gut microbiota diversity. Sci. Rep..

[15] Mahdavinia M, Keshavarzian A, Tobin MC, Landay AL, Schleimer RP (2016). A comprehensive review of the nasal microbiome in chronic rhinosinusitis (CRS). Clin. Exp. Allergy.

[16] Segura M, Calzas C, Grenier D, Gottschalk M (2016). Initial steps of the pathogenesis of the infection caused by *Streptococcus suis*: fighting against nonspecific defenses. FEBS Lett..

[17] Mou KT (2019). Shifts in the nasal microbiota of swine in response to different dosing regimens of oxytetracycline administration. Vet. Microbiol..

[18] Zeineldin M, Aldridge B, Blair B, Kancer K, Lowe J (2018). Microbial shifts in the swine nasal microbiota in response to parenteral antimicrobial administration. Microb. Pathog..

[19] Correa-Fiz F, Goncalves Dos Santos JM, Illas F, Aragon V (2019). Antimicrobial removal on piglets promotes health and higher bacterial diversity in the nasal microbiota. Sci. Rep..

[20] Slifierz MJ, Friendship RM, Weese JS (2015). Longitudinal study of the early-life fecal and nasal microbiotas of the domestic pig. BMC Microbiol..

[21] Wang X, Tsai T, Deng F, Wei X, Chai J, Knapp J (2019). Longitudinal investigation of the swine gut microbiome from birth to market reveals stage and growth performance associated bacteria. Microbiome.

[22] Frese SA, Parker K, Calvert CC, Mills DA (2015). Diet shapes the gut microbiome of pigs during nursing and weaning. Microbiome.

[23] McCormack UM, Curiao T, Buzoianu SG, Prieto ML, Ryan T, Varley P (2017). Exploring a Possible Link between the Intestinal Microbiota and Feed Efficiency in Pigs. Appl. Environ. Microbiol..

[24] Fouhse JM, Zijlstra RT, Willing BP (2016). The role of gut microbiota in the health and disease of pigs. Anim. Front..

[25] Pluske JR, Turpin DL, Kim JC (2018). Gastrointestinal tract (gut) health in the young pig. Anim. Nutr..

[26] Chen L, Xu Y, Chen X, Fang C, Zhao L, Chen F (2017). The maturing development of gut microbiota in commercial piglets during the weaning transition. Front. Microbiol..

[27] Bian G, Ma S, Zhu Z, Su Y, Zoetendal EG, Mackie R (2016). Age, introduction of solid feed and weaning are more important determinants of gut bacterial succession in piglets than breed and nursing mother as revealed by a reciprocal cross-fostering model. Environ. Microbiol..

[28] Su Y, Yao W, Perez-Gutierrez ON, Smidt H, Zhu WY (2008). Changes in abundance of *Lactobacillus* spp. and *Streptococcus suis* in the stomach, jejunum and ileum of piglets after weaning. FEMS Microbiol. Ecol..

[29] Xiang Q (2020). Early-life intervention using fecal microbiota combined with probiotics promotes gut microbiota maturation, regulates immune system development, and alleviates weaning stress in piglets. Int. J. Mol. Sci..

[30] De Witte C, Flahou B, Ducatelle R, Smet A, De Bruyne E, Cnockaert M (2017). Detection, isolation and characterization of *Fusobacterium gastrosuis* sp. nov. colonizing the stomach of pigs. Syst. Appl. Microbiol..

[31] Lorenzo de Arriba, M., Lopez-Serrano, S., Galofre-Mila, N., Aragon, V. Characterisation of *Bergeyella* spp. isolated from the nasal cavities of piglets*. Vet. J.* **234**, 1–6 (2018). 10.1016/j.tvjl.2018.01.004 29680378

[32] Wang Q, Cai R, Huang A, Wang X, Qu W, Shi L (2018). Comparison of oropharyngeal microbiota in healthy piglets and piglets with respiratory disease. Front. Microbiol..

[33] Ramayo-Caldas Y (2016). Phylogenetic network analysis applied to pig gut microbiota identifies an ecosystem structure linked with growth traits. ISME J..

[34] Yang H, Yang M, Fang S, Huang X, He M, Ke S (2018). Evaluating the profound effect of gut microbiome on host appetite in pigs. BMC Microbiol..

[35] Correa-Fiz F, Fraile L, Aragon V (2016). Piglet nasal microbiota at weaning may influence the development of Glasser's disease during the rearing period. BMC Genomics.

[36] Larsson J, Fall N, Lindberg M, Jacobson M (2016). Farm characteristics and management routines related to neonatal porcine diarrhoea: a survey among Swedish piglet producers. Acta Vet. Scand..

[37] Kongsted H, Toft N, Nielsen JP (2014). Risk factors and epidemiological characteristics of new neonatal porcine diarrhoea syndrome in four Danish herds. BMC Vet. Res..

[38] Zijlmans MA, Korpela K, Riksen-Walraven JM, de Vos WM, de Weerth C (2015). Maternal prenatal stress is associated with the infant intestinal microbiota. Psychoneuroendocrinology.

[39] Ison SH, Jarvis S, Hall SA, Ashworth CJ, Rutherford KMD (2018). Periparturient behavior and physiology: further insight into the farrowing process for primiparous and multiparous sows. Front. Vet. Sci..

[40] Jiang L, Feng C, Tao S, Li N, Zuo B, Han D (2019). Maternal imprinting of the neonatal microbiota colonization in intrauterine growth restricted piglets: a review. J. Anim. Sci. Biotechnol..

[41] Maltecca C, Bergamaschi M, Tiezzi F (2020). The interaction between microbiome and pig efficiency: a review. J. Anim. Breed. Genet..

[42] Rodríguez JM (2015). The composition of the gut microbiota throughout life, with an emphasis on early life. Microb. Ecol. Health Dis..

[43] Han GG, Lee JY, Jin GD (2018). Tracing of the fecal microbiota of commercial pigs at five growth stages from birth to shipment. Sci. Rep..

[44] Ishida S, le Tien HT, Osawa R, Tohya M, Nomoto R, Kawamura Y (2014). Development of an appropriate PCR system for the reclassification of *Streptococcus suis*. J. Microbiol. Methods.

[45] Galofre-Mila N, Correa-Fiz F, Lacouture S, Gottschalk M, Strutzberg-Minder K, Bensaid A (2017). A robust PCR for the differentiation of potential virulent strains of *Haemophilus parasuis*. BMC Vet. Res..

[46] Okura M, Lachance C, Osaki M, Sekizaki T, Maruyama F, Nozawa T (2014). Development of a two-step multiplex PCR assay for typing of capsular polysaccharide synthesis gene clusters of *Streptococcus suis*. J. Clin. Microbiol..

[47] Versalovic J, Koeuth T, Lupski JR (1991). Distribution of repetitive DNA sequences in eubacteria and application to fingerprinting of bacterial genomes. Nucleic Acids Res..

[48] Klindworth A, Pruesse E, Schweer T, Peplies J, Quast C, Horn M (2013). Evaluation of general 16S ribosomal RNA gene PCR primers for classical and next-generation sequencing-based diversity studies. Nucleic Acids Res..

[49] Bolyen E, Rideout JR, Dillon MR, Bokulich NA, Abnet CC, Al-Ghalith GA (2019). Reproducible, interactive, scalable and extensible microbiome data science using QIIME 2. Nat. Biotechnol..

[50] Callahan BJ, McMurdie PJ, Rosen MJ, Han AW, Johnson AJ, Holmes SP (2016). DADA2: high-resolution sample inference from Illumina amplicon data. Nat. Methods.

[51] Katoh K, Misawa K, Kuma K, Miyata T (2002). MAFFT: a novel method for rapid multiple sequence alignment based on fast Fourier transform. Nucleic Acids Res.

[52] Price MN, Dehal PS, Arkin AP (2010). FastTree 2–approximately maximum-likelihood trees for large alignments. PLoS ONE.

[53] Bokulich NA, Kaehler BD, Rideout JR, Dillon M, Bolyen E, Knight R (2018). Optimizing taxonomic classification of marker-gene amplicon sequences with QIIME 2's q2-feature-classifier plugin. Microbiome.

[54] McDonald D, Price MN, Goodrich J, Nawrocki EP, DeSantis TZ, Probst A (2012). An improved Greengenes taxonomy with explicit ranks for ecological and evolutionary analyses of bacteria and archaea. ISME J..

[55] Vazquez-Baeza Y, Pirrung M, Gonzalez A, Knight R (2013). EMPeror: a tool for visualizing high-throughput microbial community data. Gigascience.

[56] Okasnen, J *et al*. Vegan: Community Ecology Package. R package version 2.3-0 (2015).

[57] Anderson, M. J. A new method for non-parametric multivariate analysis of variance. *Austral. Ecol.* **26,** 32–46 (2001).

[58] Becker, R. A., Chambers, J. M., & Wilks, A. R. *The New S Language*. Wadsworth & Brooks/Cole (1988).

[59] Chambers, J. M., Cleveland, W. S., Kleiner, B., & Tukey, P. A. *Graphical Methods for Data Analysis*. Wadsworth & Brooks/Cole (1983).

[60] Murrell, P. *R Graphics*. Chapman & Hall/CRC Press (2005).

[61] Studio Team. RStudio: Integrated Development for R. RStudio, Inc., Boston, MA. https://www.rstudio.com/ (2015).

