# Supplementary material for: Feed additives for the control of post-weaning Streptococcus suis disease and the effect on the faecal and nasal microbiota
Source: Sci Rep. 2020 Nov 23;10:20354. doi: 10.1038/s41598-020-77313-6 (PMC7683732; doi:10.1038/s41598-020-77313-6)
Supplement: Supplementary file 1 — Supplementary Information [file 41598_2020_77313_MOESM1_ESM.pdf]

Feed additives for the control of post-weaning *Streptococcus suis* disease and the effect on the faecal and nasal microbiota

Florencia Correa-Fiz[1,2], Carlos Neila-Ibáñez[1,2], Sergio López-Soria[1,2], Sebastian Napp[1,2], Blanca Martinez[3], Laia Sobrevia[3], Simon Tibble[3], Virginia Aragon[1,2], Lourdes Migura-Garcia[1,2,*]

[1] IRTA, Centre de Recerca en Sanitat Animal (CReSA, IRTA-UAB), Campus de la Universitat Autònoma de Barcelona, 08193 Bellaterra, Spain

[2] OIE Collaborating Centre for the Research and Control of Emerging and Re-emerging Swine Diseases in Europe (IRTA-CReSA), Bellaterra, Barcelona, Spain

[3] ASN

Supplementary Table S1. Genera that dynamically changed over time in the faecal microbiota, detected by ANCOM performed at genus level.

OTU	W	Average D0 (%)	Average D21 (%)	Average Lys (%)	Average FA+Lys (%)	Average Control (%)	Average Amox (%)	Average FA+antiinf (%)
k__Bacteria;p__Bacteroidetes;c__Bacteroidia;o__Bacteroidales;f__;g__	157	2,071837765	16,93233179	16,95704	21,10919945	17,58985689	13,93428318	14,77146993
k__Bacteria;p__Bacteroidetes;c__Bacteroidia;o__Bacteroidales;f__[Odoribacteraceae];g__Butyricimonas	155	1,252296588	0,101504102	0,004061	0,065200006	0,353298158	0	0,068434166
k__Bacteria;p__Bacteroidetes;c__Bacteroidia;o__Bacteroidales;f__[Odoribacteraceae];g__Odoribacter	155	0,481496602	0,000567978	0,141531	0	0,003092325	0,08720253	0
k__Bacteria;p__Bacteroidetes;c__Bacteroidia;o__Bacteroidales;f__Bacteroidaceae;g__Bacteroides	154	18,3472542	0,523309389	0,193866	0,262011989	0,471389105	0,10904523	0,147281344
k__Bacteria;p__Bacteroidetes;c__Bacteroidia;o__Bacteroidales;f__RF16;g__	152	0,00398216	0,558231472	1,439814	0,418219542	0,120293403	0,66291316	0,478096548
k__Bacteria;p__Bacteroidetes;c__Bacteroidia;o__Bacteroidales;f__Rikenellaceae;g__	143	4,763222382	0,121284483	0,016043	0,187632303	0,057390429	0,00951936	0,247160408
k__Bacteria;p__Bacteroidetes;c__Bacteroidia;o__Bacteroidales;f__Rikenellaceae;g__Alistipes	143	1,57296345	0,003276922	0,003835	0,016056916	0	0	0
k__Bacteria;p__Firmicutes;c__Bacilli;o__Lactobacillales;f__Lactobacillaceae;g__Lactobacillus	141	0,595457018	0,003949696	0,737307	0	0	0,5348408	0,003638128
k__Bacteria;p__Firmicutes;c__Clostridia;o__Clostridiales;f__Clostridiaceae;g__	137	0,029866065	3,670848572	0,230919	3,821476807	7,750763073	0,24099144	2,731368528
k__Bacteria;p__Firmicutes;c__Clostridia;o__Clostridiales;f__Lachnospiraceae;g__Coprococcus	133	0,0191026	0,376728957	0	0,251022922	0,295339875	0	0,361585281
k__Bacteria;p__Firmicutes;c__Clostridia;o__Clostridiales;f__Ruminococcaceae;g__Faecalibacterium	132	0,033731918	0,889054169	25,94345	0,82087381	0,66479276	24,26856312	1,011755585
k__Bacteria;p__Firmicutes;c__Erysipelotrichi;o__Erysipelotrichales;f__Erysipelotrichaceae;g__RFN20	131	0,307351701	3,439530674	0,000588	5,27132532	1,971538181	0,00187935	2,998104881
k__Bacteria;p__Fusobacteria;c__Fusobacteriia;o__Fusobacteriales;f__Fusobacteriaceae;__	127	0,845150143	0,000150295	0,10688	0	0	0,58520335	0
k__Bacteria;p__Fusobacteria;c__Fusobacteriia;o__Fusobacteriales;f__Fusobacteriaceae;g__Fusobacterium	127	1,449095584	0,004430373	0,29229	0	0,021846692	0,79570149	0
k__Bacteria;p__Lentisphaerae;c__[Lentisphaeria];o__Victivallales;f__Victivallaceae;g__	125	0,362552648	0,01133828	0,984271	0,02958947	0,014327898	1,09791812	0,007513744
k__Bacteria;p__Proteobacteria;c__Deltaproteobacteria;o__Desulfovibrionales;f__Desulfovibrionaceae;g__Desulfovibrio	125	0,156004059	0,008624806	3,868721	0,00442675	0,023306923	4,3283741	0,009333686
k__Bacteria;p__Proteobacteria;c__Gammaproteobacteria;o__Enterobacteriales;f__Enterobacteriaceae;__	124	1,615778057	0,001269779	5,322212	0,004696794	0	7,47340731	0,001525121
k__Bacteria;p__Proteobacteria;c__Gammaproteobacteria;o__Pasteurellales;f__Pasteurellaceae;g__Actinobacillus	122	0,193055685	0	4,21451	0	0	3,30867063	0
k__Bacteria;p__Spirochaetes;c__Spirochaetes;o__Spirochaetales;f__Spirochaetaceae;g__Treponema	120	0,186646456	3,003130745	0,046083	4,013621727	4,601746286	0,07468926	2,662325745
k__Bacteria;p__Tenericutes;c__Mollicutes;o__RF39;f__;g__	119	0,831047941	2,294823504	0	2,420327483	1,943293284	0	1,284402293

Supplementary Table S2. Genera that dynamically changed over time in the nasal microbiota, detected by ANCOM performed at genus level.

OTU	W	Average D0 (%)	Average D21 (%)	Average Lys (%)	Average FA+Lys (%)	Average Control (%)	Average Amox (%)	Average FA+antiinf (%)
k__Bacteria;p__Actinobacteria;c__Actinobacteria;o__Actinomycetales;f__Actinomycetaceae;g__Actinomyces	258	0,08676455	0,005083262	0,000710656	0,013421553	0,007060162	0,002462052	0,001761887
k__Bacteria;p__Actinobacteria;c__Actinobacteria;o__Actinomycetales;f__Micrococcaceae;g__Arthrobacter	279	0,1350221	0,00807043	0	0,00406067	0,03104778	0,00524371	0
k__Bacteria;p__Actinobacteria;c__Coriobacteriia;o__Coriobacteriales;f__Coriobacteriaceae;__	250	0,0302162	0,09079637	0,07802891	0,14153117	0,05906288	0,08815634	0,08720254
k__Bacteria;p__Actinobacteria;c__Coriobacteriia;o__Coriobacteriales;f__Coriobacteriaceae;g__Olsenella	268	0,0081424	0,14181441	0,05254158	0,19386571	0,2991961	0,05442341	0,10904523
k__Bacteria;p__Bacteroidetes;c__[Saprospirae];o__[Saprospirales];f__Chitinophagaceae;g__	312	0,09201343	0,86742428	1,615809	1,43981437	0,37665078	0,24193407	0,66291317
k__Bacteria;p__Bacteroidetes;c__Bacteroidia;o__Bacteroidales;f__[Odoribacteraceae];g__Butyricimonas	254	0,22173555	0,01068995	0,01465782	0,01604308	0,00997432	0,0032552	0,00951935
k__Bacteria;p__Bacteroidetes;c__Bacteroidia;o__Bacteroidales;f__[Odoribacteraceae];g__Odoribacter	300	0,35259375	0,00154348	0	0,00383481	0,00086774	0,00301486	0
k__Bacteria;p__Bacteroidetes;c__Bacteroidia;o__Bacteroidales;f__[Paraprevotellaceae];g__CF231	250	0,19519877	0,66097531	0,72213964	0,73730701	0,53437885	0,77621027	0,53484081
k__Bacteria;p__Bacteroidetes;c__Bacteroidia;o__Bacteroidales;f__Bacteroidaceae;g__Bacteroides	263	4,79565869	0,44403518	0,43091307	0,23091929	1,13032831	0,18702377	0,24099145
k__Bacteria;p__Bacteroidetes;c__Bacteroidia;o__Bacteroidales;f__BS11;g__	306	0,16809131	8,32E-05	0	0	0,00041588	0	0
k__Bacteria;p__Bacteroidetes;c__Bacteroidia;o__Bacteroidales;f__Prevotellaceae;g__Prevotella	244	7,40099352	23,0149483	21,9845443	25,9434512	19,6171859	23,2609969	24,2685631
k__Bacteria;p__Bacteroidetes;c__Bacteroidia;o__Bacteroidales;f__Rikenellaceae;g__Alistipes	245	0,07193883	0,00207165	0,00505465	0,00058783	0,00193199	0,00090446	0,00187935
k__Bacteria;p__Bacteroidetes;c__Flavobacteriia;o__Flavobacteriales;f__[Weeksellaceae];g__Bergeyella	306	22,5087523	0,32983082	0,44278647	0,10688035	0,35091564	0,1633683	0,58520336
k__Bacteria;p__Cyanobacteria;c__4C0d-2;o__YS2;f__;g__	251	0,09410558	0,51095041	0,32672096	0,29228954	0,53947232	0,60056772	0,7957015
k__Bacteria;p__Firmicutes;c__Clostridia;o__Clostridiales;f__;g__	255	1,92633003	1,16663683	1,11517768	0,98427112	1,54658981	1,08922744	1,09791811
k__Bacteria;p__Firmicutes;c__Clostridia;o__Clostridiales;f__Clostridiaceae;g__	239	1,28261549	3,72534404	1,8147339	3,86872148	3,82417333	4,7907174	4,32837409
k__Bacteria;p__Firmicutes;c__Clostridia;o__Clostridiales;f__Lachnospiraceae;g__	283	5,79192666	5,74876804	5,42663496	5,32221246	4,96239715	5,55918834	7,47340731
k__Bacteria;p__Firmicutes;c__Clostridia;o__Clostridiales;f__Lachnospiraceae;g__Blautia	285	0,49510569	3,48384506	3,43589239	4,21450985	3,37192114	3,08823129	3,30867063
k__Bacteria;p__Firmicutes;c__Clostridia;o__Clostridiales;f__Lachnospiraceae;g__Butyrivibrio	295	0	0,04987088	0,03034334	0,04608285	0,04178547	0,0564535	0,07468927
k__Bacteria;p__Firmicutes;c__Clostridia;o__Clostridiales;f__Lachnospiraceae;g__Clostridium	312	0,98333592	0,00283125	0,00956418	0	0,00081573	0,00377636	0
k__Bacteria;p__Firmicutes;c__Clostridia;o__Clostridiales;f__Lachnospiraceae;g__Coprococcus	259	0,45297757	1,62544837	1,47683052	1,62258311	1,44538314	1,68635667	1,89608841
k__Bacteria;p__Firmicutes;c__Clostridia;o__Clostridiales;f__Lachnospiraceae;g__Dorea	263	0,42512245	1,73002336	1,72926043	1,79904287	1,75709071	1,69987802	1,66484476
k__Bacteria;p__Firmicutes;c__Clostridia;o__Clostridiales;f__Lachnospiraceae;g__Lachnospira	292	0,04170612	0,42226193	0,21335164	0,38855312	0,2221907	0,55572308	0,7314911
k__Bacteria;p__Firmicutes;c__Clostridia;o__Clostridiales;f__Lachnospiraceae;g__Pseudobutyrivibrio	244	0	0,0300666	0,03041612	0,03680215	0,01310864	0,02050756	0,04949854
k__Bacteria;p__Firmicutes;c__Clostridia;o__Clostridiales;f__Lachnospiraceae;g__Roseburia	260	0,66146287	3,32512705	2,08645566	3,0393766	2,50582352	4,30483823	4,68914124
k__Bacteria;p__Firmicutes;c__Clostridia;o__Clostridiales;f__Peptostreptococcaceae;g__	307	0,01234636	0,16839679	0,14931073	0,17388528	0,1819246	0,17039339	0,16646996
k__Bacteria;p__Firmicutes;c__Clostridia;o__Clostridiales;f__Peptostreptococcaceae;g__Peptostreptococcus	298	0,77636172	0,02210738	0,02062576	0,01303877	0,05704915	0,01083299	0,00899022
k__Bacteria;p__Firmicutes;c__Clostridia;o__Clostridiales;f__Ruminococcaceae;g__Faecalibacterium	299	0,25603705	3,1391728	2,33849594	3,71633385	2,82028737	3,20740605	3,61334077
k__Bacteria;p__Firmicutes;c__Clostridia;o__Clostridiales;f__Ruminococcaceae;g__Gemmiger	283	0,39307407	2,92234498	2,75270501	3,29849003	2,8755572	2,41805114	3,26692153
k__Bacteria;p__Firmicutes;c__Clostridia;o__Clostridiales;f__Ruminococcaceae;g__Ruminococcus	252	0,90388817	2,89212099	2,54692408	2,67442783	2,74456234	3,04833994	3,44635075
k__Bacteria;p__Firmicutes;c__Erysipelotrichi;o__Erysipelotrichales;f__Erysipelotrichaceae;g__Bulleidia	289	0,09869264	0,96545174	0,8213804	1,16256146	1,11104405	0,83945558	0,8928172
k__Bacteria;p__Fusobacteria;c__Fusobacteriia;o__Fusobacteriales;f__Fusobacteriaceae;g__	306	1,55564484	0,01723308	0,02494449	0,02532848	0,0209895	0,01382151	0,00108144
k__Bacteria;p__Fusobacteria;c__Fusobacteriia;o__Fusobacteriales;f__Fusobacteriaceae;g__Fusobacterium	304	8,93213246	0,7933709	0,18485684	0,09444426	3,57094399	0,05970769	0,05690173
k__Bacteria;p__Proteobacteria;c__Gammaproteobacteria;o__Aeromonadales;f__Succinivibrionaceae;g__Anaerobiospirillum	295	0,25823436	0,00034945	0	0,00074195	0,00078886	0,00021644	0
k__Bacteria;p__Proteobacteria;c__Gammaproteobacteria;o__Aeromonadales;f__Succinivibrionaceae;g__Succinivibrio	287	0,00688992	0,11257452	0,05055346	0,14531967	0,14771935	0,04482969	0,17445041
k__Bacteria;p__Proteobacteria;c__Gammaproteobacteria;o__Pasteurellales;f__Pasteurellaceae;g__Mannheimia	262	0,00101542	0,05653723	0,12174531	0,05764223	0,04768124	0,03294752	0,02266987
k__Bacteria;p__Tenericutes;c__Mollicutes;o__Anaeroplasmatales;f__Anaeroplasmataceae;g__	249	0,0014255	0,0175155	0,00579908	0,01391173	0,01925265	0,02389566	0,02471837

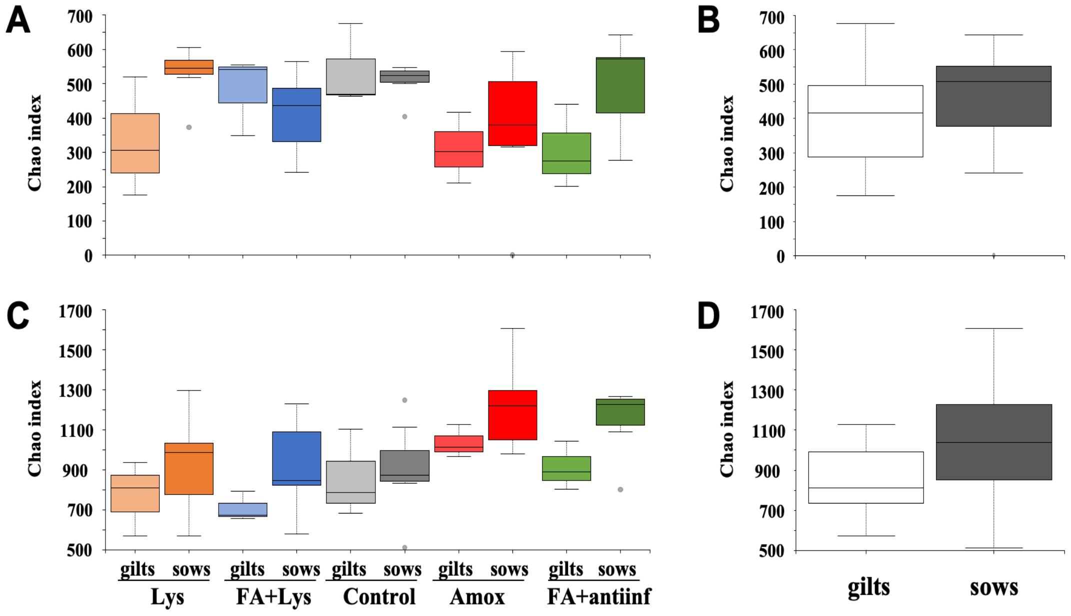

Supplementary Figure S1. Richness of faecal and nasal microbiota is different depending on the treatment group. Richness was estimated using Chao's index at D21 on faecal (A) or nasal (C) samples for the treatment groups considering the parity of the dams. Also, the richness was estimated for faecal (B) or nasal (D) microbiota grouping animals regarding the parity of their dams without considering the group they belonged. The dotted lines represent standard deviation and outliers are indicated with white circles. The treatment groups were: Lys, lysozyme; FA+ lys, medium chain fatty acids and lysozyme; C, control, with no additives; Amox, amoxicillin; FA+antiinf: medium chain fatty acids and a natural antiinflammatory; each group was splitted according the parity of their dams: gilts (first delivery) or sows (more than 1 delivery).

A

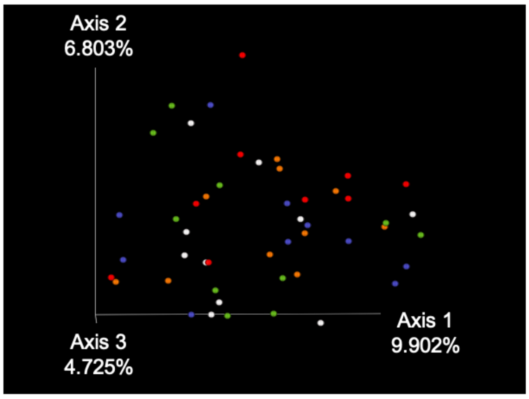

Group 1	Group 2	*P* value
Lys	Lys + FA	0.844
	Control	0.064
	Amox	0.120
	FA + antiinf	0.101
Lys + FA	Control	0.139
	Amox	0.081
	FA + antiinf	0.200
Control	Amox	0.089
	FA + antiinf	0.206
Amox	FA + antiinf	0.169

B

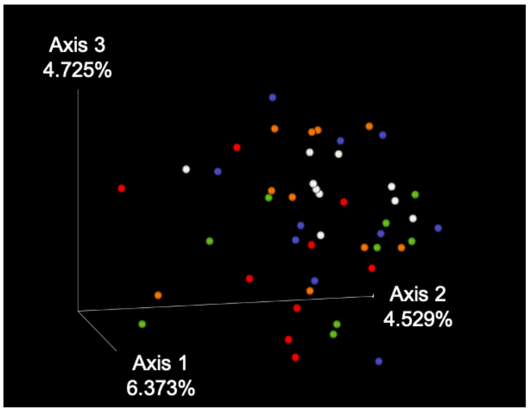

Group 1	Group 2	*P* value
Lys	Lys + FA	0.784
	Control	0.125
	Amox	0.027
	FA + antiinf	0.077
Lys + FA	Control	0.140
	Amox	0.044
	FA + antiinf	0.168
Control	Amox	0.034
	FA + antiinf	0.133
Amox	FA + antiinf	0.075

Supplementary Figure S2. Beta diversity analysis of the faecal microbiota composition in different treatment groups. Beta diversity analysis with Bray Curtis' (A) or Jaccard (B) distances of the faecal microbiota composition among samples collected at D21 for different treatment groups. The significance estimated with PERMANOVA pairwise analysis (at 999 permutations) for each metric, is shown in the right panel indicating the *P* values. The treatment groups were: Lys, lysozyme; FA+ lys, medium chain fatty acids and lysozyme; C, control, with no additives; Amox, amoxicillin; FA+antiinf: medium chain fatty acids and a natural anti-inflammatory. The principal axes are shown with the percentage of variation explained between brackets. *** stands for *P* value < 0.001.

A

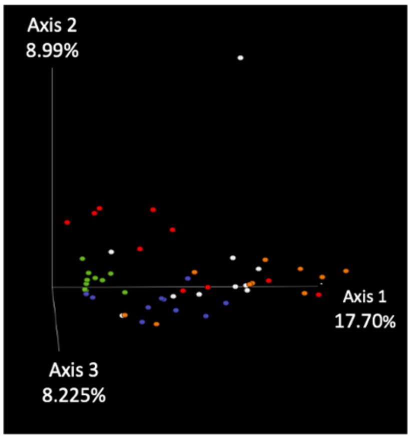

Group 1	Group 2	*P* value
Lys	Lys + FA	0.001
	Control	0.006
	Amox	0.001
	FA + antiinf	0.001
Lys + FA	Control	0.002
	Amox	0.001
	FA + antiinf	0.001
Control	Amox	0.009
	FA + antiinf	0.001
Amox	FA + antiinf	0.001

B

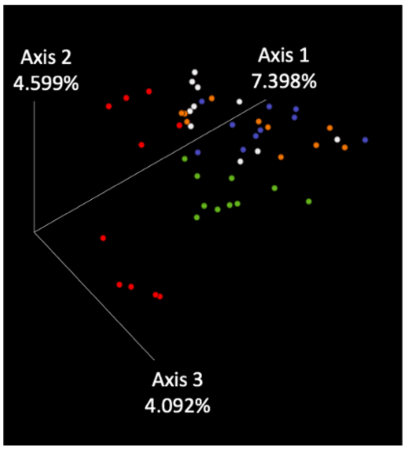

Group 1	Group 2	*P* value
Lys	Lys + FA	0.003
	Control	0.003
	Amox	0.001
	FA + antiinf	0.001
Lys + FA	Control	0.001
	Amox	0.001
	FA + antiinf	0.001
Control	Amox	0.001
	FA + antiinf	0.001
Amox	FA + antiinf	0.001

Supplementary Figure S3. Beta diversity analysis of the nasal microbiota composition in different treatment groups. Beta diversity analysis with Bray Curtis' (A) or Jaccard (B) distances of the faecal microbiota composition among samples collected at D21 for different treatment groups. The significance estimated with PERMANOVA pairwise analysis (at 999 permutations) for each metric, is shown in the right panel indicating the *P* values. The treatment groups were: Lys, lysozyme; FA+ lys, medium chain fatty acids and lysozyme; C, control, with no additives; Amox, amoxicillin; FA+antiinf: medium chain fatty acids and a natural anti-inflammatory. The principal axes are shown with the percentage of variation explained between brackets. *** stands for *P* value < 0.001.
